# Evaluating AI decision tools in Ecuador’s courts: efficiency, consistency, and uncertainty in legal judgments

**DOI:** 10.3389/frai.2025.1688209

**Published:** 2025-11-06

**Authors:** Eliana Rodríguez-Salcedo, Carlos Martínez-Bonilla, Betty Pérez-Mayorga, Mónica Salame-Ortiz, Pamela Armas-Freire, Anita Espín-Miniguano, Eulalia Pino-Loza

**Affiliations:** Faculty of Law, Universidad Técnica de Ambato, Ambato, Ecuador

**Keywords:** advanced analytical methods, algorithmic governance, artificial intelligence, judicial decision-making, natural language processing, procedural efficiency, uncertainty in legal systems

## Abstract

This study explores the impact of AI-based decision support tools on judicial performance in Ecuador, a context characterized by institutional uncertainty and procedural inefficiencies. It assesses whether such tools improve efficiency, consistency, and the normative quality of legal reasoning in judicial decisions. A mixed-methods approach was applied to analyze fifty court cases before and after AI implementation. Quantitative analysis used *t*-tests, Levene’s test, and Mann–Whitney U test to evaluate procedural duration and inter-rater agreement, while natural language processing techniques, including topic modeling (LDA) and sentiment analysis (VADER), assessed changes in semantic structure and argumentation. In parallel, a content analysis of twelve policy and regulatory documents was conducted to examine changes in algorithmic governance discourse. The results show a statistically significant reduction in case resolution time (−23.5 days), an increase in inter-evaluator consistency (Cohen’s kappa from 0.65 to 0.80), a shift toward more neutral-technical language, and greater density of legal citations. Mentions of governance principles such as transparency and accountability also increased. These findings indicate that AI-based tools, when used as assistive systems, can enhance judicial decision-making in uncertain environments without displacing human deliberation. While the study provides robust initial evidence, its exploratory sample and reliance on interpretable NLP techniques reflect the constraints of a low-resource judicial context and highlight avenues for future research. This research contributes to the literature on advanced analytical methods for institutional decision-making under legal and epistemic uncertainty.

## 1 Introduction

Contemporary judicial systems constitute complex socio-technical structures, in which legal regulations, human actors, information flows and changing institutional environments interact (Beim and Rader, 2019). This complexity is traversed by high levels of structural uncertainty, which are manifested in the unpredictability of resolution times, interpretative variability between judges and the difficulty in ensuring traceability in decisions (De Cruz, 2024). Such uncertainty compromises not only the efficiency of the judicial system, but also its legitimacy, transparency, and perception of fairness (Segura, 2023; Villalba, 2020).

These challenges are intensified in judicial systems with limited infrastructure, regulatory fragmentation, and low levels of digitalization, as is the case in several Latin American countries (Juárez-Merino, 2025; Morić et al., 2025). In this context, artificial intelligence (AI) has emerged not only as a promising set of tools, but as a computational framework capable of operationalizing institutional uncertainty and benchmarking judicial performance (Sanchez and García, 2023). Techniques such as Bayesian networks, machine learning algorithms, and natural language processing (NLP) pipelines enable the large-scale analysis of legal and procedural data, providing measurable indicators of efficiency, consistency, and argumentative density (Adriano Fabre et al., 2024; Aldave Orzaiz, 2021; Morales Cáceres, 2021). These methods contribute to reducing decisional uncertainty by quantifying resolution times, mapping normative references, and standardizing semantic structures in judicial discourse (López Vega et al., 2023; Luna Salas et al., 2023).

However, the adoption of AI in justice also poses ethical, normative, and epistemological risks. If adequate governance mechanisms are not implemented, algorithmic systems can amplify historical biases, compromise principles of due process, and affect fundamental rights (Bedê and Campos, 2024; Hedler, 2024a). For this reason, organizations such as the OECD and the European Commission have issued guidelines on the responsible use of AI, promoting principles such as algorithmic transparency, institutional accountability, and meaningful human oversight (OCDE, 2025). These guides are especially relevant for countries in digital transition such as Ecuador, where the implementation of AI must be articulated with regulatory frameworks aligned with international standards (Morte Ferrer, 2021; Rivera, 2023).

**Figure 1:**
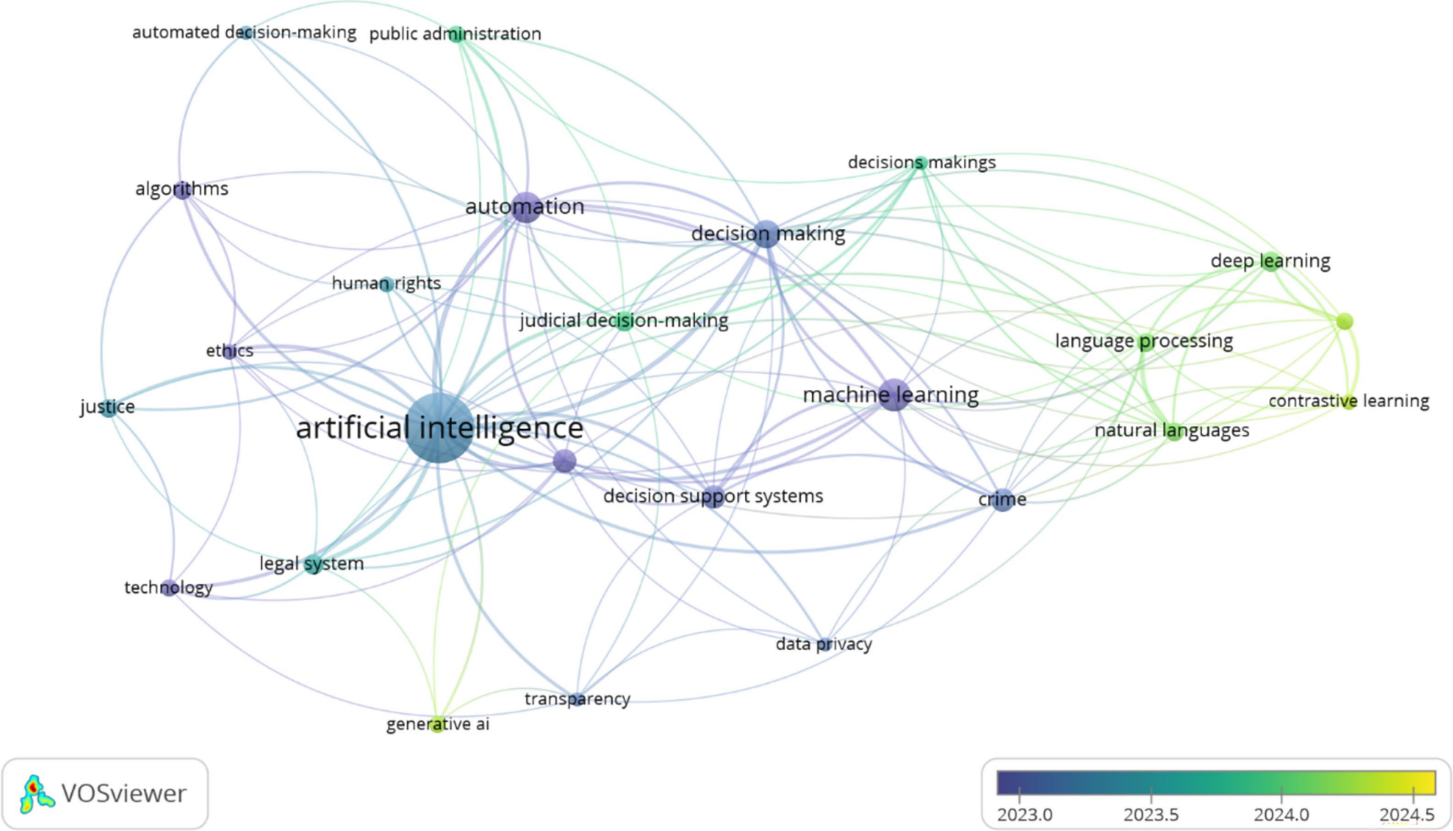
Keyword co-occurrence map generated with VOSviewer, based on Scopus-indexed literature (2020–2025). The size of each node reflects its frequency; colors represent average publication year.

In order to visualize how the literature connects AI, judicial decision-making, and normative concerns, a bibliometric co-occurrence analysis was conducted using Scopus-indexed publications from 2020 to 2025. As shown in Figure 1, thematic clusters converge around key concepts such as artificial intelligence, decision-making, automation, and machine learning, while also connecting with issues of ethics, data privacy, and judicial fairness. This network highlights the multidimensional and interdisciplinary nature of the field, as well as its temporal evolution and increasing complexity. The bibliometric dataset supporting Figure 1 has been deposited in Zenodo (Rodríguez-Salcedo et al., 2025; Doi: 10.5281/zenodo.17186752) to ensure transparency and replicability.

This article introduces and validates a computational pipeline for judicial analytics, integrating statistical inference, semantic modeling, sentiment analysis, and documentary evaluation to measure the effects of AI-based decision support in Ecuadorian courts. Ecuador provides a critical testbed as a low-resource, high-uncertainty environment, where the transferability of AI pipelines remains underexplored. Unlike previous studies concentrated in Brazil or Colombia, this research offers one of the first systematic evaluations of AI’s institutional effects in Ecuador.

The study is framed as a methodological contribution to the field of decision-making under uncertainty, positioning AI as an assistive but auditable technology. Beyond the Ecuadorian case, it aims to contribute to broader debates on algorithmic governance, digital justice, and institutional resilience in Latin America, in alignment with the United Nations Sustainable Development Goals (SDG 9: Innovation and Infrastructure; SDG 16: Peace, Justice and Strong Institutions).

This research is part of the funded project “La Inteligencia Artificial (IA) y su aplicación en la Educación y profesión del Derecho” [*Artificial Intelligence (AI) and its Application in Legal Education and the Legal Profession*], supported by the Universidad Técnica de Ambato, which seeks to foster innovative applications of AI in legal education and professional practice.

## 2 Theoretical framework

### 2.1 Applications of AI in Latin American courts: empirical experiences

AI adoption in Latin American courts has advanced in Brazil, Colombia, Argentina and Chile, where pilot systems demonstrate both opportunities and risks. Initiatives such as Prometea in Argentina, which reduced processing times, and the Santiago Declaration in Chile, which emphasizes ethical AI governance, illustrate regional leadership but also highlight persistent issues such as the digital divide and algorithmic bias (Juárez-Merino, 2025).

In Colombia, the PretorIA system in the Constitutional Court uses big data and blockchain to locate legal information, improving efficiency but raising due process concerns (Botero Chica et al., 2024). Comparative analyses of PretorIA and Brazil’s Victor show that these tools assist with synthesis and prediction but cannot substitute judicial deliberation; scholars recommend magistrate participation in design and oversight (Calderon-Valencia et al., 2021).

In Brazil, large-scale systems are more prevalent. INACIA, based on large language models, automates evaluation tasks in the Court of Auditors, showing high correlation with human reasoning but requiring stronger traceability frameworks (Pereira et al., 2025). LegalAnalytics, which classifies appeals in the Federal Supreme Court, incorporates explainable AI (LIME) to ensure transparency and has been validated by experts (Resck et al., 2025). Other proposals, such as redesigning the order-for-payment procedure with AI, aim to relieve judicial overload without undermining due process (Pereira Campos, 2024).

Overall, these experiences confirm that AI can optimize workloads and improve access to legal information, but robust methodological validation and governance frameworks remain necessary to ensure accountability.

### 2.2 Judicial prediction and algorithmic consistency evaluation

A growing body of research applies predictive modeling to anticipate judicial outcomes and evaluate consistency. In Brazil, deep learning architectures such as Hierarchical Attention Networks have been used to predict results in criminal cases, achieving both accuracy and explainability by identifying the linguistic features most influential in judicial reasoning (Bertalan and Ruiz, 2024). Similarly, large-scale experiments with more than 4,000 cases obtained F1-scores above 80%, confirming the feasibility of outcome prediction at scale (Lage-Freitas et al., 2022).

Another line of work focuses on precedent retrieval and consistency checking. Comparative studies of more than a hundred algorithmic configurations demonstrate that granular textual embeddings and summarization techniques improve jurisprudential coherence (Mentzingen et al., 2024). Subsequent approaches integrating summarization with language models such as ADA have produced scalable solutions that balance accuracy with computational cost, making them viable for resource-constrained judicial environments (Mentzingen et al., 2025).

These studies underscore that predictive analytics and precedent retrieval are not only technically feasible but also replicable across judicial systems, provided that methods are adapted to local data availability and governance requirements.

### 2.3 Regulatory frameworks, ethical risks and legal uncertainty

While AI offers efficiency gains, its integration into judicial decision-making raises persistent ethical and regulatory concerns. Studies highlight risks such as bias amplification, opacity, and erosion of judicial independence if algorithms are used beyond assistive functions (Bedê and Campos, 2024; Melo, 2024).

Regional initiatives, such as Brazil’s Justice 4.0 program, promote centralized supervision of algorithms rather than automation of rulings, reflecting tensions between innovation and doctrinal safeguards (Hedler, 2024a). Comparative analyses of European and Latin American frameworks emphasize the need for precautionary principles, transparency, and explainability (Hedler, 2024b).

Overall, consensus is emerging that AI in law must remain assistive, auditable, and embedded within normative frameworks that preserve due process and human oversight (De Sanctis, 2021).

### 2.4 Advanced analytical methods for judicial decision-making in uncertain contexts

AI research in judicial analytics increasingly combines quantitative inference with semantic and normative modeling. Common techniques include NLP pipelines, Latent Dirichlet Allocation (LDA), and lexicon-based sentiment analysis tools such as VADER. These allow the extraction of argumentative patterns and discursive tone from judicial texts (Barik and Misra, 2024; Jelodar et al., 2019). Although more recent approaches based on transformer architectures (e.g., BERT and its derivatives) achieve higher performance in topic modeling and sentiment analysis, LDA and VADER remain widely used in legal informatics due to their interpretability, lower computational requirements, and transparency, qualities that are especially relevant in judicial contexts where explainability is critical (Adriano Fabre et al., 2024; Hedler, 2024b).

In parallel, statistical tools such as t-tests, Mann–Whitney’s U, and Cohen’s *κ* are applied to measure procedural efficiency and inter-evaluator agreement, providing replicable metrics of judicial performance (Alves, 2017; Molina, 2023). Qualitative approaches such as thematic coding and normative benchmarking against international standards complement these methods, enabling fuzzy inference under legal uncertainty (López Vega et al., 2023; Luna Salas et al., 2023).

This hybrid toolkit reflects not only a systemic approach but also a pragmatic balance: combining computational, statistical, and normative layers that are feasible in resource-constrained environments, while recognizing the potential of more advanced NLP models for future research.

### 2.5 Justification of the study

Despite regional progress, gaps remain in evaluating AI in judicial systems from an integrated perspective. Most studies concentrate on large, digitized courts (Brazil, Colombia), while smaller and less digitized systems, such as Ecuador’s, remain underexplored.

Empirical evidence on the actual impact of AI on efficiency, argumentative coherence, and governance principles is fragmented, and there is no consensus on normative criteria to guide implementation. This creates risks for legitimacy and sustainability of judicial AI. This study addresses these gaps by providing empirical evidence from Ecuador, a low-resource and high-uncertainty environment, and by explicitly testing whether methods that are explainable and computationally accessible (LDA, VADER, traditional statistics) can deliver meaningful insights under such conditions. This design allows assessing the transferability and robustness of AI methods in under-documented contexts.

### 2.6 Research objectives

Based on the gaps identified in the literature, this study seeks to comprehensively address the impact of AI on judicial systems in Latin America, particularly in Ecuador. The central objective of this study is to evaluate the impact of AI-based decision support systems in Ecuadorian courts, considering their influence on procedural efficiency, decisional coherence and the normative quality of judicial reasoning, as well as on the principles of institutional governance within the framework of due process. The following specific objectives are proposed from this objective: (a) to determine whether the implementation of artificial intelligence in Ecuadorian courts significantly improves procedural efficiency, measured through the average time of resolution of cases; (b) to analyze whether the use of artificial intelligence in the judicial process increases the interevaluative coherence and improves the argumentative quality of decisions, measured by Cohen’s *κ* coefficient, thematic analysis of legal reasoning and density of legal citations; (c) To explore the impact of artificial intelligence on the discursive and institutional frameworks of judicial governance, through the analysis of documentary content and the tonality of discourse in judicial decisions.

Accordingly, three hypotheses are formulated: The implementation of AI in Ecuadorian courts will significantly reduce the average time for resolving cases compared to the period prior to its adoption (H_1_); the use of AI in Ecuadorian courts will be associated with greater inter-evaluator coherence and an improvement in the normative quality of judicial reasoning, reflected in an increase in Cohen’s κ coefficient, greater emphasis on principles of procedural fairness, and a higher density of legal citations (H_2_); the adoption of AI in Ecuadorian courts will significantly increase the presence of governance principles (transparency, accountability, and human supervision) in normative documents and modify the tone of judicial discourse towards more neutral or positive positions (H_3_).

## 3 Materials and methods

This study followed a convergent mixed-methods design, integrating quantitative evaluation of judicial performance with qualitative-documentary analysis of AI governance. The approach combines advanced decisional analytics, thematic modeling, sentiment analysis, statistical inference, and normative coding, suitable for environments of high institutional uncertainty.

### 3.1 Quantitative design: evaluation of judicial performance

The quantitative component compared judicial outcomes before and after AI adoption in a sample of 50 cases (25 pre-implementation, 25 post-implementation), selected through purposive sampling and matched by jurisdiction and procedural typology. This number of cases is statistically sufficient to apply mean-comparison tests, but it should be understood as an exploratory design given the constraints on access to judicial files in Ecuador (Alves, 2017; Molina, 2023).

To evaluate procedural efficiency, resolution times were compared using Student’s t-test (with Levene test for variance homogeneity) (Bahamón et al., 2023) and Mann–Whitney U for non-normal distributions (Lascano-Arias et al., 2025). Both tests were applied with a 95% confidence level, ensuring robustness even with a small sample size, as recommended in studies of judicial analytics under constrained conditions (López Vega et al., 2023).

Inter-evaluator coherence was estimated using Cohen’s *κ* coefficient and paired decisions by human judges with and without algorithmic support.

Natural language processing (NLP) techniques were incorporated, including Latent Dirichlet Allocation (LDA) for thematic modeling and VADER for sentiment analysis. LDA was configured with five topics based on coherence score optimization, while VADER was selected for its transparency and interpretability in legal discourse analysis. Although more advanced transformer-based methods (e.g., BERT) have been shown to outperform traditional approaches, explainable models such as LDA and VADER remain preferable in legal studies where interpretability and reproducibility are critical (Adriano Fabre et al., 2024; Hedler, 2024b). These tools were used to extract argumentative structures and discursive polarity.

Likewise, the density of legal citations per 1,000 words was computed as a proxy for depth of legal reasoning. All computational procedures were developed in Python (v. 3.11.2), using Scikit-learn, Gensim, NLTK, and Pandas (Mehare et al., 2023).

### 3.2 Qualitative design: documentary and regulatory analysis

In parallel, a systematic qualitative content analysis was carried out on 12 official documents, including technical manuals, regulatory standards and public policies issued before and after the implementation of the AI system. These documents were selected through a comprehensive search in the official repositories of the Ecuadorian judiciary and related government institutions, applying inclusion criteria of relevance to AI governance and publication between 2018 and 2025.

Coding was based on the European Commission’s Guidelines for Trustworthy AI, focused on three critical dimensions: (i) algorithmic transparency, (ii) institutional accountability, and (iii) meaningful human intervention. The coding process was carried out by two independent researchers, applying a double-reading and cross-checking procedure. The reliability of the intercoder was evaluated using the Krippendorff *α*, with values ≥ 0.80 in all categories, which ensures the interpretative soundness of the results. This step anchors empirical findings within auditable international governance frameworks.

### 3.3 Triangulation and validation

Triangulation integrated efficiency, coherence, and semantic results with regulatory analysis, enabling cross-validation and a systemic mapping of AI’s institutional impact. This methodological triangulation enhances internal validity and provides a stronger foundation for generalizability, even in low-resource judicial contexts where longitudinal data are scarce (Segura, 2023).

### 3.4 Ethics and confidentiality

All the judicial files used were anonymized in accordance with the provisions of the Organic Law on the Protection of Personal Data of Ecuador in 2021 (Hernández Alvarado et al., 2023). No personal data or sensitive information that would allow the identification of natural or legal persons was included.

### 3.5 Statement on the use of generative artificial intelligence

During the preparation of this study, generative artificial intelligence tools were used only for writing, grammar and spelling correction tasks. In no case were these tools used for data generation, methodological design, or the elaboration of the substantive content of the article. Its use was strictly auxiliary and did not affect the scientific or academic integrity of the manuscript.

## 4 Results

This section reports the outcomes of the computational pipeline, integrating statistical, semantic, and governance indicators. Results are organized along six analytical dimensions: efficiency, coherence, reasoning, tone, normative density, and governance. All findings reflect a before–after comparative design, allowing assessment of AI’s impact on judicial decision-making under conditions of institutional uncertainty.

### 4.1 Procedural efficiency

The results in Table 1 show a statistically significant reduction in the length of judicial prosecution following the introduction of AI. The mean time decreased from 72.4 days (SD = 13.2) in the pre-IA period to 48.9 days (SD = 10.7) in the post-IA period. Levene’s test (*p* = 0.10) confirmed the homogeneity of variances, allowing the use of a t-test for independent samples [t(48) = 5.24, *p* < 0.001]. The 95% confidence interval for the difference was [18.6, 28.4] days, suggesting a substantial impact on procedural efficiency attributable to the algorithmic system. Given the limited sample size (*N* = 50), these findings should be considered exploratory and interpreted with caution, although they align with trends reported in other judicial analytics studies.

**Table 1:** Comparing case processing time metrics before and after AI deployment.

Period	M (days)	SD	Levene test	*T*-test	CI (95%)
Pre-AI (*N* = 25)	72.4	13.2	0.10		
Post-AI (*N* = 25)	48.9	10.7		*p* < 0,001	[18.6,28.4]

M, mean; SD, standard deviation; CI, confidence interval; *N*, number of cases. The Levene test confirms the homogeneity of variances (*p* = 0.10).

### 4.2 Inter-evaluator coherence

Table 2 shows Cohen’s *κ* coefficients obtained for paired decisions in five pairs of cases, both before and after AI assistance. A generalized increase in agreement between evaluators was observed. The average value of κ increased from 0.65 (interpreted as substantial agreement) to 0.80 (near-perfect agreement), according to the Landis and Koch classification. This improvement suggests that AI not only streamlines procedures, but also standardizes criteria, promoting greater decisional uniformity among judges. While greater uniformity enhances coherence, it also raises questions about potential reduction of interpretive diversity, a point further developed in the discussion section.

**Table 2:** Summaries of Cohen’s *k* for paired decision before and after AI implementation.

Case pair ID	*k* (pre-AI)	*k* (post-AI)	Λ*k*
1	0.62	0.78	+0.16
0.2	0.58	0.74	+0.16
3	0.67	0.82	+0.15
4	0.70	0.85	+0.15
5	0.63	0.79	+0.16
In general	0.65	0.80	+0.15

*κ, Cohen’s kappa coefficient; Λk, change in kappa value between pre- and post-AI conditions.*

### 4.3 Thematic modeling of legal reasoning

**Figure 2:**
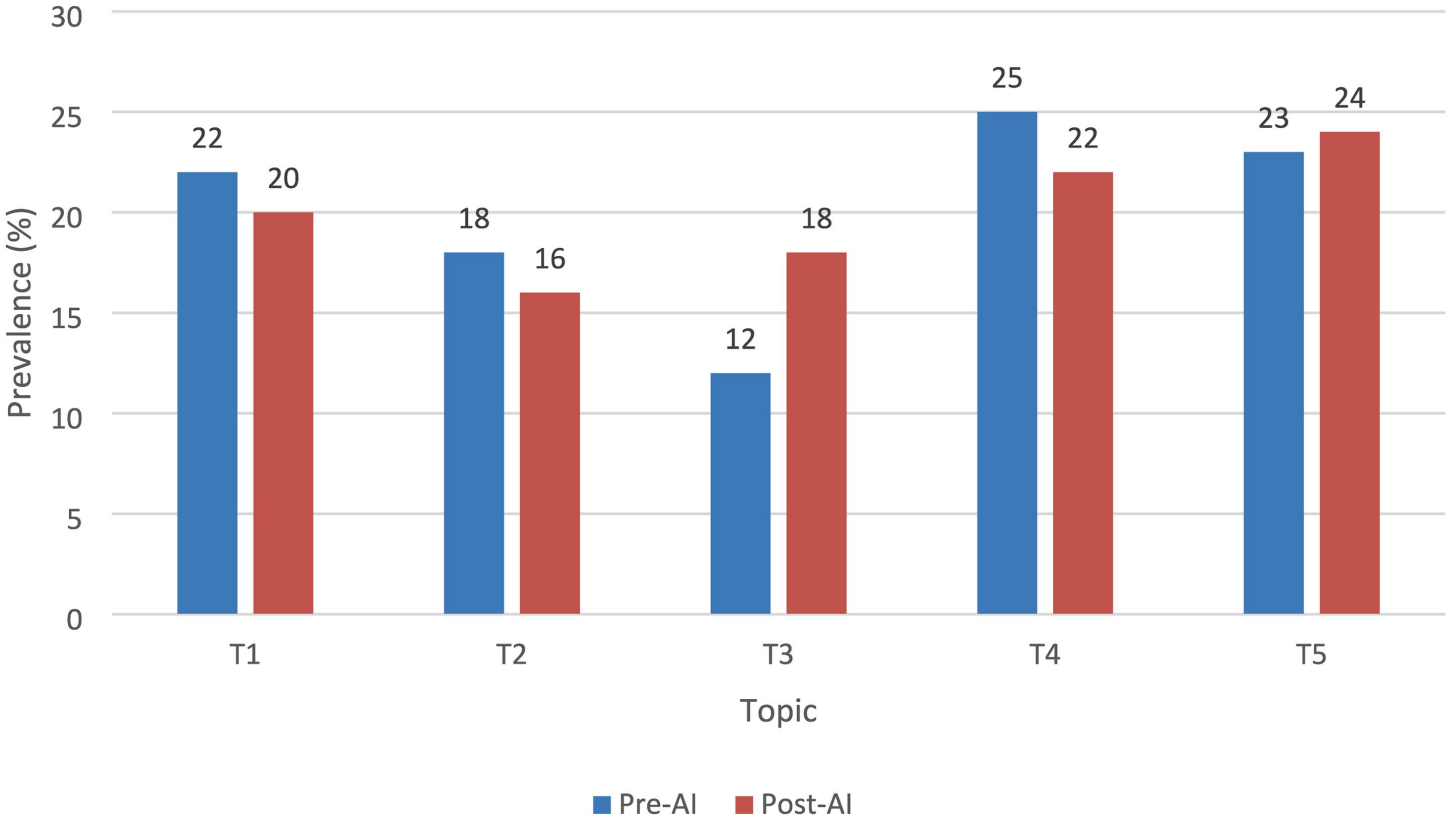
The Top 5 LDA Themes, Pre-AI vs. Post-AI. T1, evidence and procedural risk; T2, appeals and judicial accuracy; T3, equity, impartiality and rights; T4, sentencing and efficiency; T5, jurisdiction and monitoring.

Figure 2 graphically represents the five main themes identified by LDA (Latent Dirichlet Allocation) modeling in the analyzed statements, comparing their distribution before and after the implementation of AI. The results show a relevant semantic change, especially in the increase of the T3 topic, associated with “equity, impartiality and rights,” whose proportion grew by six percentage points in the post-AI period. This shift suggests a greater normative orientation towards fundamental procedural principles following the introduction of the algorithmic system, while T1 shifted toward more technical and risk-related terms, and T4 and T5 incorporated optimization and automation language.

Table 3, on the other hand, details the keywords that make up each of the five topics extracted, allowing us to observe how the semantic content of judicial decisions varies depending on automated assistance. For example, in T1 a shift from “witnesses” and “load” to “evidence,” “algorithm” and “risk” is observed, reflecting a greater presence of automated technical-legal language.

**Table 3:** Summaries of Cohen’s *k* for paired decision before and after AI implementation.

Theme	Pre-AI keywords	Post-AI keywords
T1	Evidence, witnesses, charging	Evidence, algorithm, risk
T2	Judgment, appeal, precedent	Judgment, model, accuracy
T3	Equity, impartiality, rights	Fairness, transparency, procedure
T4	Sentence, punishment, retribution	Judgment, optimization, efficiency
T5	Jurisdiction, competence	Jurisdiction, automation, monitoring

Themes (T1–T5) were extracted using Latent Dirichlet Allocation (LDA) topic modeling applied to judicial decisions pre- and post-AI implementation.

### 4.4 Argumentative tone

Sentiment analysis, in Table 4, reveals a significant change in the discursive tonality: from a slightly negative mean (−0.05) before the AI, to a neutral or slightly positive tone (+0.02) after its implementation (*U* = 230, *p* = 0.03). This finding suggests that the language of judicial decisions became more technical and less evaluative, due to the intervention of algorithmic systems that modulate the writing style.

**Table 4:** Average sentiment scores in court texts before and after AI.

Theme	M	SD	Mann–Whitney U	*p* value
Pre-AI	−0.05	0.12	230	0.03
Post-AI	+0.02	0.10		

M, mean number of legal citations per 1,000 words; SD, standard deviation. *p* value, probability value indicating statistical significance.

### 4.5 Density of legal citations

Table 5 shows the average number of normative citations per 1,000 words in the judgments of both periods, together with the statistical significance of the comparison. The results indicate a significant increase in regulatory density following the implementation of AI. The average went from 15.3 to 18.7 citations per 1,000 words [t (48) = 2.15, *p* < 0.05]. This reflects a greater technical structuring and legal reference in algorithmically assisted decisions, contributing to the argumentative soundness of the rulings.

**Table 5:** Average citations per 1,000 words before and after AI.

Theme	M	SD	*p*-value
Pre-AI	15.3	4.8	
Post-AI	18.7	5.1	p < 0.05

M, mean number of legal citations per 1,000 words; SD, standard deviation. *p*-value, probability value from independent-samples *t*-test.

### 4.6 Institutional governance

Table 6 presents the relative frequency of thematic codes in relevant normative documents, before and after the introduction of AI in the judicial environment. Coding of 12 policy documents showed notable increases in transparency (65 → 92%), accountability (48 → 85%), and oversight (55 → 88%). The consistency of the coding process was validated with a Krippendorff *α* ≥ 0.80 in all categories. This confirms that algorithmic integration is paralleled by regulatory adaptation, reinforcing governance dimensions essential for trustworthy AI.

**Table 6:** Frequency of thematic codes in AI-related policy documents.

Theme	Pre-AI documents (%)	Post-AI documents (%)
Transparency	65	92
Responsibility	48	85
Human supervision	55	88

## 5 Discussion

The general objective of this study was to evaluate the impact of AI-based decision support systems in Ecuadorian courts, considering their influence on procedural efficiency, decisional coherence, normative quality of judicial reasoning, and the principles of institutional governance under the framework of due process. The findings indicate that algorithmic assistance produced measurable improvements, particularly in reducing case resolution times, increasing inter-evaluator consistency, and reinforcing transparency and accountability in governance frameworks. This evidence supports the claim that AI can contribute to mitigating structural uncertainty in judicial systems (De Cruz, 2024; Segura, 2023), while situating Ecuador as a valuable case for expanding debates on digital justice beyond the more commonly studied contexts of Brazil and Colombia (Morić et al., 2025).

### 5.1 Procedural efficiency (H1)

H1 predicted that the implementation of AI would significantly reduce case resolution times. The results confirmed this hypothesis: case processing times decreased by an average of 23.5 days, supporting the claim that AI can enhance procedural efficiency under conditions of resource scarcity. This outcome is consistent with Juárez-Merino (2025), who documented a comparable reduction with Prometea in Argentina, and with Botero Chica et al. (2024), who reported improvements with PretorIA in Colombia.

Unlike prior research focused on institutional or systemic aggregates, the present study provides micro-level evidence that links algorithmic tools with performance indicators at the case level. From a decisional perspective, this acceleration mitigates temporal and logistical uncertainty, strengthening both predictability and confidence in judicial operations. Nevertheless, the limited sample size (n = 50) requires interpreting these improvements as exploratory rather than conclusive, echoing recommendations by Alves (2017) for careful statistical framing in judicial analytics.

### 5.2 Inter-evaluator coherence and regulatory quality (H2)

H2 proposed that AI would improve inter-evaluator coherence and enrich the normative quality of legal reasoning. The increase in Cohen’s *κ* from substantial to near-perfect agreement validates H_2_ and aligns with findings by Lage-Freitas et al. (2022), who noted that predictive models reduce interpretative variability.

This improvement suggests that AI acted as a stabilizer of judicial interpretation, promoting greater consistency without eliminating judicial autonomy. The thematic analysis revealed a stronger orientation towards principles of fairness and due process, complemented by a higher density of legal citations. Together, these indicators reflect a qualitative enrichment of legal reasoning, consistent with Mentzingen et al. (2025), who emphasized that hybrid models strengthen jurisprudential coherence.

However, decisional uniformity also presents risks: while greater *κ* values increase reliability, they may reduce interpretive diversity and flexibility, as noted by Hedler (2024b). This tension underscores the need for AI to be designed as an assistive, not substitutive, system. These results therefore challenge more critical perspectives, such as those of Bedê and Campos (2024), by showing that AI in this context operated as an auxiliary tool rather than a substitute for deliberation.

### 5.3 Institutional governance and due process (H3)

H3 predicted that AI adoption would reinforce governance principles such as transparency, accountability, and human oversight. The documentary analysis confirmed this hypothesis, showing notable increases in references to these principles across normative documents. This is consistent with international frameworks such as the EU’s Ethical Guidelines for Trustworthy AI and the OECD’s AI Principles, which emphasize precaution and oversight as prerequisites for legitimacy.

In addition, sentiment analysis revealed a shift towards more neutral or slightly positive tones, suggesting a less emotional and more standardized discursive style. This resonates with observations by Adriano Fabre et al. (2024) on the linguistic modulation capacity of AI systems, and it represents an underexplored contribution to Latin American debates. These findings support the idea that AI not only affects judicial outputs (rulings) but also reshapes the institutional and communicative structures that sustain them, thus reducing both normative and discursive uncertainty in judicial governance (Melo, 2024).

### 5.4 Critical considerations and scope of the study

Although the results are promising, several limitations must be acknowledged. First, the small case sample (*n* = 50) restricts generalizability and should be understood as a pilot design. Second, the use of LDA and VADER, while interpretable and transparent, is less advanced than current transformer-based models (e.g., BERT), which may offer deeper semantic insights but at the cost of reduced explainability (Adriano Fabre et al., 2024). Third, the short observation window prevents evaluation of long-term sustainability. Fourth, case selection may involve bias. Finally, the lack of cross-national comparison limits the scope of inference.

These constraints align with concerns raised by De Sanctis (2021) and Melo (2024), who caution against uncritical automation. The present findings should thus be understood as exploratory, highlighting the potential of AI to support, but not replace, judicial deliberation.

Future research should expand to larger and longitudinal datasets, incorporate bias detection and fairness metrics, and assess the effects of algorithmic systems on public trust in justice (Hedler, 2024a). Comparative studies between countries at different stages of digitalization would also help to establish the transferability of AI pipelines across judicial environments. Such steps would enable the development of auditable and transferable models of algorithmic governance aligned with global standards of trustworthy AI.

## 6 Conclusion

This study evaluated the impact of AI-based decision support systems in Ecuadorian courts, focusing on procedural efficiency, decisional coherence, argumentative quality, and institutional governance. The results provide empirical evidence that algorithmic assistance can reduce structural uncertainty in judicial systems, producing tangible gains in efficiency, interpretative consistency, and normative density. Specifically, H1 was supported by the significant reduction in case resolution times, H2 by the improvement in inter-evaluator coherence and enrichment of legal reasoning, and H3 by the reinforcement of governance principles in normative documents.

Unlike previous literature concentrated in Brazil or Colombia, this research offers the first systematic evidence for Ecuador, thereby broadening comparative perspectives on digital justice in Latin America. The findings confirm that AI can operate as an auxiliary tool that strengthens judicial performance without displacing deliberation or undermining judicial autonomy. At the same time, the observed increase in decisional uniformity highlights both benefits (greater reliability and stability) and potential risks (reduced interpretive diversity), underscoring the importance of designing AI systems as assistive rather than substitutive mechanisms.

At the institutional level, the increased emphasis on transparency, accountability, and human oversight in policy documents demonstrates that technological innovation was accompanied by governance reinforcement. This alignment with global frameworks such as the OECD Principles and the EU Ethical Guidelines reinforces the legitimacy of AI use in justice, provided that precautionary and ethical safeguards are maintained.

Nevertheless, the scope of this study is limited by its small sample size, the short observation period, and the absence of cross-jurisdictional comparisons. The reliance on interpretable but less advanced NLP techniques (LDA and VADER) also reflects methodological trade-offs between transparency and performance, a limitation that future research should address by comparing explainable and transformer-based models (e.g., BERT, RoBERTa) in judicial contexts. These constraints underscore the need for longitudinal studies with larger datasets, fairness and bias audits, and analyses of AI’s impact on public trust and legitimacy.

In conclusion, this study demonstrates that AI has the potential to become a supportive mechanism for enhancing predictability, coherence, and transparency in Latin American judicial systems. However, its contribution will depend on robust governance, ethical safeguards, and continuous empirical evaluation, particularly in low-resource environments where replicability and explainability are as critical as performance.

## Data Availability

The raw data supporting the conclusions of this article will be made available by the authors, without undue reservation.
